# A Typology of Poles’ Attitudes toward COVID-19 during the First Wave of the Pandemic

**DOI:** 10.3390/ijerph18042002

**Published:** 2021-02-19

**Authors:** Rafał Boguszewski, Marta Makowska, Monika Podkowińska

**Affiliations:** Department of Sociology, Institute of Sociological Sciences and Pedagogy, Warsaw University of Life Sciences, Nowoursynowska 166 St., 02-787 Warsaw, Poland; rafal_boguszewski@sggw.edu.pl (R.B.); monika_podkowinska@sggw.edu.pl (M.P.)

**Keywords:** Poland, SARS-CoV-2, attitudes, government restrictions, public health

## Abstract

(1) Objective: To explore Poles’ attitudes during the first wave of COVID-19 pandemic in 2020 as a contribution toward the creation of effective health policies. (2) Method: Computer-assisted web interviewing (CAWI) was used to survey a sample of 1001 Poles selected using quota sampling. (3) Results: Using cluster analysis, three types of attitudes were distinguished, people being classified as “involved” (48.1%), “cautious” (27.4%), or “indifferent” (24.6%). The result of greatest interest was the absence of any attitude indicating an extremely dismissive posture toward COVID-19. Three logistic regression analyses, comparing people displaying each attitude with those comparing the other two attitudes combined, showed that an involved attitude was likely to be associated with being female, being in a poorer financial situation, but having relatively high life satisfaction. A cautious attitude was more likely to appear in places with fewer residents and among people in a favorable financial situation, and that an indifferent attitude was more likely to be associated with being male and having lower life satisfaction. (4) Conclusions: The attitudes identified may help to explain why, during the spring of 2020, the virus was spreading slightly more slowly, and on a narrower scale, in Poland than in other countries.

## 1. Introduction

The SARS-CoV-2 virus, a new coronavirus first detected in December 2019 in Wuhan, China, causes COVID-19 disease and has significantly changed the functioning of societies in many countries, including Poland. The first case of COVID-19 was officially confirmed in Poland on March 4. On March 11, when 31 cases of infection had been recorded, the Polish government began to announce restrictions to curb the spread of the virus. On March 13, Ministry of Health regulations introduced, inter alia, movement restrictions and a stay-at-home policy [1], and on the 20th of March an epidemic was officially declared in Poland [2]. The strictest restrictions were in force between the 1st and 20th of April. In addition to the movement restrictions, other restrictions included: the closing of educational facilities; a ban on using parks, forests, beaches, boulevards, etc.; significant restrictions on access to grocery stores and the functioning of shopping malls and large-format DIY stores; a suspension of hairdressing and cosmetic businesses; the closing of restaurants; shutting down of passenger-carrying air traffic; the closure of international rail traffic and domestic rail traffic restrictions. On April 16, an additional obligation for people to cover their mouths and noses was introduced [3], but from April 20, a gradual “defrosting of the economy” began, in which some restrictions were eased, although people still had to take many precautions.

Compared to other countries, during the spring of 2020, Poland had relatively few cases, although its infection rate was still higher than in some of its neighboring countries. In mid-June, around 820 people per million inhabitants had been diagnosed as infected, as compared with the following numbers of cases per million in other countries: USA, 6900; Belgium, 5200; UK, 4400; Italy, 3900; but only 280 in neighboring Slovakia [4]. The present article is based on data collected at the start of the pandemic (during the first wave in Poland, which occurred in the spring of 2020).

The SARS-CoV-2 vaccine was not yet available at the time of the study, and vaccination programs have only just commenced at the time of writing. Because of the limited number of effective medical interventions available, many countries have found it necessary to implement drastic preventive measures, such as people being asked to socially distance and infected people being instructed to quarantine once identified. Such measures have aimed to limit the number of people infected at any one time, so that the countries’ healthcare systems do not become overloaded [5]: so-called “flattening the curve”, i.e., causing the number of cases to fall from one day to the next [6]. It is likely that Poland experienced a smaller number of cases at the beginning of the pandemic relative to many other countries because of the very early use of a strong lockdown mechanism; such a policy was more popular in the countries of Eastern and Central Europe than in the countries of Western Europe [7]. But other factors, such as people’s feelings of a strong ethical responsibility for the seriously ill and elderly, and the high degree of social intergenerational solidarity that exists in Poland, might also have been beneficial [7]. The pandemic and its associated restrictions have had a great impact on the daily routines of Poles: many people have started to work from home, schools have been closed, contacts with other people have become less frequent, etc. Some Polish researchers have studied these changes. For example, one study found a negative but nonsignificant relationship between Poles’ use of public transport and the number of new cases of COVID-19 [8], and another study discussed students’ acceptance of higher education’s shift to distance education modes of delivery [9].

As in other countries [10,11], in Poland the pandemic has negatively affected many people’s sense of well-being, there being a heightened threat of unemployment and fears of losing family members [12]. Wearing a mask has become a common obligation in many countries, and it has been noted that prevailing sociocultural contexts need to be examined, and values such as solidarity and communal safety need to be emphasized if attempts to persuade people to wear masks are to be successful [13]. A 2020 study comparing Poland with China identified a large difference in face mask-wearing: 35% of Poles vs. 96% of Chinese people [14]. This is perhaps not surprising given that face mask usage was not encouraged in Poland at the time data were collected (at the beginning of the pandemic) for this study, although it was encouraged in China. Furthermore, mask usage was common in Asia before the COVID-19 pandemic due to their past respiratory virus epidemics experience. There is also a greater cultural emphasis on interdependence in Asian countries [13], and it has been shown that e-government has a strong effect on people’s attitudes toward quarantine in China [15]. To date, it appears that Polish researchers have not investigated whether local government can influence Poles’ attitudes and decision-making processes via e-government platforms, but it can be assumed that this influence is significantly smaller than in China.

Social psychology has a long history of studying why people obey authority [16]. This subject becomes extremely important in the context of a pandemic, where people are required to adhere to a governing authority’s recommendations. In attempting to explain why some countries have achieved higher levels of adherence to COVID-19-related lockdowns than others, it is necessary to look at differences in historical contexts and contemporary leadership between countries [17]. It is also necessary to take cognizance of the fact that differences within countries are likely to exist. For example, Italian research has shown that most well-educated people, women, middle-aged people, health workers, and people living in Southern Italy are the most likely to comply with quarantine orders. They have also found that attitudinal factors matter: people with greater anxiety and at greater risk of contracting COVID-19 are also more likely to comply with quarantine orders [18].

In our currently reported analysis, we focus on Poles’ attitudes toward COVID-19 as one possible factor explaining the comparatively slow spread of the virus in Poland during the first wave of the pandemic. We expected to observe diverse attitudes and the emergence of extreme attitudes as the views of Polish people and societal influences were considered to be polarized at the time the research was conducted [19].

Our article aims to identify and characterize Poles’ attitudes toward the COVID-19 pandemic. The prevalence of different types of attitudes within the Polish population is discussed, and the socio-demographic characteristics associated with these differing attitudes are identified. Traditionally, three attitudinal components have been distinguished: affective, cognitive, and behavioral components [20,21,22]. The affective component is a necessary component for any attitude, and involves the extent of a person’s positive or negative emotional feelings toward an attitude object, e.g., the new coronavirus. The cognitive component concerns a person’s perceptions and beliefs regarding an attitude object. Finally, the behavioral component relates to a person’s behaviors, behavioral intentions, or behavioral desires toward an attitude object. The cognitive and behavioral components are not necessary for the emergence of an attitude. Attitude identification is often an important element of the work of people involved in disease prevention and health promotion [22] because this helps to provide an understanding of people’s perceptions of an issue, assists in the identification of barriers, and gives an idea of the extent of people’s support for various health-related activities. This type of research can help identify different groups with different needs and identify the different approaches necessary to help people switch toward healthier behaviors [23,24]. During the COVID-19 pandemic, identification of people’s attitudes is highly important: it can show the extent to which people are reacting differently to the crisis and help us to understand why some people are more likely than others to follow guidelines aimed at limiting the spread of the virus. It is necessary to monitor attitudes in different local and cultural contexts since people’s attitudes and the factors determining them may differ across such contexts. Studies concentrating on attitudes toward COVID-19 of populations of various specific countries have already been published [25,26,27,28], and other studies have also focused on different social groups, e.g., health professionals [29,30,31] and people with certain illnesses [32,33]. The amount of research on the topic is growing quickly, and this research is valuable, especially in the context of the need for public health programs to combat misinformation, stigmatization, and fears about COVID-19 [34]. Attitudinal data can help to focus such efforts on the correct groups of people. The main contribution of our research is to ascertain Polish people’s attitudes toward the pandemic during its first wave. Such research is crucial for future studies that might seek to understand the different social and cultural determinants of different nations’ initial responses to the pandemic.

## 2. Materials and Methods

The currently discussed data were collected in a survey conducted between the 14th and 20th of April 2020, using computer-assisted web interviewing (CAWI) of a sample of 1001 respondents. Quota sampling was used to ensure an appropriate representation of Polish society in terms of gender (2 groups), age (5 groups), and size of the locality of residence (4 groups). In addition, it was ensured that the sample was representative in terms of respondents’ province (Polish administrative subdivision; 16 groups) and educational level (2 groups: higher and other). The sample was selected from the SW online panel administered by the SW Research company—the leading online panel research company in Poland (Appendix A contains details of the exact distribution of respondents’ socio-demographic characteristics). All respondents took part in the study voluntarily and received an appropriate small amount of remuneration in accordance with the operating procedures of the SW Research agency. Polish regulations did not require the consent of an ethics committee for the study described in the article to be undertaken.

The questionnaire used in the survey was purposely designed by the authors for use in the present study. It contained 28 questions, including 5 substantive multi-choice questions, 1 filter question, 4 questions involving 34 attitudinal statements to which respondents indicated their degree of affirmation or disaffirmation using a 5-point Likert-scaled response format, and 18 socio-demographic questions. Multivariate analysis was performed on the responses to the Likert-scaled items in order to distinguish people’s attitudes towards the coronavirus pandemic. These items were designed to tap the three traditional attitudinal dimensions: affect, cognition, and behavior. Thus, they concerned emotions, knowledge or beliefs, and behavior relating to the pandemic.

To ensure the questionnaire’s content validity, other experts in social science methodology were consulted and the questionnaire was piloted. Given that the study was confined to Poland, there were no validation requirements with respect to translation or multicultural issues that would have arisen in an international study. It was not possible to perform full validation procedures due to the study’s time-critical nature. The questionnaire and study data set can be found at the figshare repository: https://figshare.com/articles/Poland_-_COVID-19/12547337 (accessed on 14 February 2021). The mean time taken to complete the survey was 10 min and 45 s, and the median was 8 min and 37 s. Responses were analyzed using SPSS 26. Analyses focused mainly on boundary distributions, crosstabulations, cluster analysis (the latter being performed using the k-means method to capture multidimensional dependencies and relationships by reducing observations to latent attitudinal dimensions), and logistic regression.

The cluster analysis assigned people to groups on the basis of their attitudes to the COVID-19 pandemic and its effects in a manner whereby people inside each group were similar to each other, and each group was as dissimilar to other groups as much as possible. Respondents were grouped according to their Likert-scaled affirmation/disaffirmation of the statements reflecting the cognitive, affective, and behavioral attitudinal components relating to the COVID-19 pandemic (individual answers were assigned numerical values from –2 to +2). Cluster analysis was used to analyze the attitudinal data, as this technique is often used in health promotion work because it is useful for identifying groups that are the most appropriate targets for health campaigns [35].

## 3. Results

An initial cluster analysis reduced the 34 statements tapping the three attitudinal components to a set of 25 statements that divided respondents into three groups (this division gave the most meaningful and interpretable results: statements having the same values for the final cluster centers for the three groups identified were not included). After defining the number of statements (25) and the number of clusters (3), further k-means clustering was used to separate respondents into individual clusters. Respondents were grouped into three clusters based on the similarity of their answers. By examining the contents of the statements most closely identified with each cluster, the three clusters were defined as follows: (1) indifferent, (2) involved, and (3) cautious. Distinguishing a larger number of clusters gave ambiguous results that were difficult to interpret. The final cluster centers are tabulated in Appendix B, and the detailed percentage distributions of affirmative answers for specific types of attitudes are shown in Table 1. See Appendix C for detailed breakdowns of responses to the individual statements included in the cluster analysis.

Based on the distributions of affirmative answers in individual clusters, we characterized the nature of the groups distinguished by the analysis and formed our typology of attitudes, as follows:

- Focus 1—indifferent: people with this attitude were not afraid for their health or that of their loved ones (affective component); they were indifferent to the restrictions introduced (cognitive component); they followed the recommendations selectively and mitigated them (behavioral component).
- Focus 2—involved: people with this attitude were concerned about their health and the health of their loved ones (affective component); they almost uncritically supported the restrictions introduced and wished them to be maintained (cognitive component); they followed the recommendations quite closely (behavioral component).
- Focus 3—cautious: people displaying this attitude were afraid for their own health and the health of their loved ones, and were more afraid than others of losing their job and a deterioration in their financial situation (affective component); perhaps in connection with these fears, they believed that some of the restrictions introduced were too strict and did not want them to be prolonged, not least because of the negative effects on the economy (cognitive component); they followed the government’s recommendations more than indifferent people, but not as rigorously and uncritically as people in the involved group (behavioral component).

The analysis did not reveal any attitude characterized by an extreme disregard for the pandemic situation and disparagement of the governmental restrictions, and it is important to note that increasing the number of clusters did not result in the isolation of any such extreme attitude. Examining respondents’ answers to individual statements (see Appendix C) showed that the percentage of people who consistently discredited the pandemic and its associated restrictions was around 5% during April 2020.

The cluster analysis showed that the largest group of adult Poles—almost half of all respondents—had an involved attitude (48.1%) and were afraid for mainly health and existential reasons. Consistent with this, these people strictly adhered to all the guidelines. The other two groups were of roughly the same size. A little more than a quarter of people were cautious (27.4%), and, while they were afraid for more material and professional reasons, they still complied with most guidelines. Almost the same percentage of people were indifferent (24.6%), feeling less stressed by the virus and being more liberal in complying with restrictions, although they did not underestimate their importance.

There were some slight but significant differences in the socio-demographic profiles of people displaying the three different attitudes (see Table 2).

As can be seen from Table 2, one of the socio-demographic variables that most strongly (and significantly) differentiated the three attitudinal groups was gender. Women displayed an involved attitude more often than men (53.9 vs. 41.6%) and an indifferent attitude less often (19.5 vs. 30.1%). However, age was not significantly associated with attitude type, although it is worth noting that there was a slight tendency whereby, relative to younger people, older people were more likely to show concerns relating to the coronavirus pandemic and conscientiously follow all governmental recommendations—57.1% of respondents aged 60+ were in the involved group, whereas only 46.5% of people in the youngest age group (18–29) were in this attitudinal cluster.

The relationship between attitudes and education was nonsignificant after applying the Holm–Bonferroni correction method of controlling for inflated type I error. In general, a higher level of education was associated with greater involvement. In general, a higher level of education was associated with greater involvement. For Poles with a higher education, 53.5% were in the involved group, but only 43.3% of people in the least educated category (primary, lower secondary, and vocational) were in this group. On the other hand, the percentages of people within each educational level falling into the indifferent group were similar (23.9 to 28.3%).

People’s place of residence (in terms of its population size) did not significantly differentiate their attitudes. Nevertheless, it is useful to note that 47.8% of residents of small towns and 52.2% of people living in the largest cities displayed an involved attitude. Also, rural inhabitants were slightly less involved (41.7%), and more of these people (33.8%) had a cautious attitude than was the case for people living in other places. But the percentages of people residing in each type of place in the indifferent group were similar (21.9 to 27.0%).

Self-assessed health was related to age: the older the people, the worse was their health rating (Cramer’s V = 0.21, *p* < 0.01). However, while age did not significantly differentiate people with respect to their attitudes, people’s self-declarations concerning their health did, although the effect size was small (Cramer’s V = 0.10). People declaring their health status to be bad were clearly more committed to complying with the government’s guidelines (56.1% of these people were in the involved attitudinal group) than those who rated their health as very good (41.3% of such people were in the involved attitudinal group). The percentages of people in each health category that displayed an indifferent attitude ranged from 20.2% (for those assessing their health as good) to 31.6% (for those saying their health was moderate).

Attitudes differed significantly according to frequency of participation in religious practices (Cramer’s V = 0.126, *p* < 0.001). An involved attitude was the most common attitude displayed across the whole sample (48.05% of people displaying this attitude), but this attitude was less prevalent among the most religious people (24.5%), a cautious attitude being the most common in this group (51%). In addition, people’s financial situations differed significantly across attitudes (Cramer’s V = 0.112, *p* = 0.0001), and a cautious attitude was the most common attitude among the most financially secure people (43.2%), but the least common among the least financially secure people (21.1%). This situation was reversed for the involved attitude, which was the most common attitude among the least financially secure (42.1%), but the least common attitude among the most financially secure (25.0%).

The level of life satisfaction was not significantly associated with the type of attitude shown. Although people who were satisfied with their life were slightly more likely to have an involved attitude than those who were dissatisfied (50.1 vs. 41.7%) and less likely to have an indifferent attitude (22.5 vs. 29.4%), these differences were not statistically significant.

A propensity for risk-taking (measured by people’s choices between whether they would prefer to obtain a lower amount of money with certainty or obtain twice as much money by correctly predicting a coin toss result) was another variable for which there were no differences in attitude. Finally, although the association was significant, attitudes varied only slightly according to people’s party political preferences, which is a variable that usually strongly differentiates Poles’ everyday attitudes and opinions. Here, supporters of the ruling party were more likely to have an involved attitude (53.9%), but the proportion of supporters of the main opposition party having this attitude was only slightly lower (48.4%).

To test whether the different socio-demographic characteristics were independently predictive of attitudes, three single-stage binary logistic regression analyses were performed comparing people displaying each attitude with those not displaying the attitude (i.e., people displaying the other two attitudes combined). The results of these analyses are shown in Table 3. Chi-square tests for each analysis showed that in each case the models including the socio-demographic characteristics were significantly better than constant only models: for analysis 1 (indifferent vs. others) χ^2^(9) = 23.05, *p* = 0.006, Nagelkerke R^2^ = 0.034; for analysis 2 (involved vs. others) χ^2^(9) = 48.25, *p* <0.001, Nagelkerke R^2^ = 0.063; for analysis 3 (cautious vs. others) χ^2^(9) = 25.70, *p* = 0.037, Nagelkerke R^2^ = 0.037.

Summarizing the results of the analyses in Table 3 with respect to the independent predictivity of socio-demographic variables, it can be concluded that: an indifferent attitude toward the pandemic was more likely to occur among men and people with lower life satisfaction; an involved attitude was more likely to occur among women, and people in poorer financial situations but with higher life satisfaction; a cautious attitude was a feature of inhabitants of smaller places and people in more favorable financial circumstances.

## 4. Discussion

Various types of plague have been experienced by people for centuries. Up to the beginning of the last century, various infectious diseases were the leading cause of mortality and the cause of approximately 50% of deaths [36]. The implementation of various public health protection measures (sewage installations, the development of clean drinking water systems, and the introduction of vaccinations and antimicrobials) has caused mortality rates attributable to these diseases to fall to only a few percentage points in advanced countries. In the mid-twentieth century, this situation gave politicians and scientists the illusory impression that infectious diseases were under human control. However, the following decades have seen the return of viruses that had theoretically been eradicated, and new viruses are emerging. Examples of the latter include AIDS, Ebola, SARS, MERS, Dengue, Chikungunya, and Zika [37]. Each of these viruses has had huge social consequences, both with respect to changes in behavior and people’s perceptions of threat. Apart from AIDS, none of these viruses have presented a danger in Poland and therefore, even where world bodies announced mobilization to fight them, the daily experiences of most Poles were not changed. Compared to certain other nations (e.g., China and the Republic of Korea), when SARS-CoV-2 appeared, because of a lack of experience, Poland (along with many other western nations) lacked any strongly developed ideas of how to respond to a pandemic. Political decisions had to be taken quickly, and Polish people’s adherence to the order to “stay at home” was extremely high. American data indicate that in States with “official orders”, people’s mobility was more reduced in comparison to States without them [38]. Official orders also influenced Poles; immediately after the introduction of restrictions, Polish citizens significantly reduced their movement—more than Italians and considerably more than the British. Geolocation data indicated that the distance the average Pole traveled fell by half within a few days of the government introducing the first restrictions [39]. Our research and the attitudes identified seems to be in line with these observations. All three of the attitudes we distinguished using cluster analysis indicated a high degree of compliance with the rather strict restrictions introduced. Other nationwide studies carried out during the same period confirm this impression. Most Poles have been shown to treat the coronavirus pandemic as something unique and unprecedented [40], and almost 70% of respondents in one study were found to fear infection [41]. People’s anecdotal observations of others and reports of their own behavior indicate that such concerns have resulted in almost universal compliance with the government’s restrictions on leaving home and contact with other people [40]. In addition, one study has shown a correlation between the spread of COVID-19 and the number of online searches for personal protective equipment and hand hygiene preparations [42].

The great degree of social mobilization among Poles, apparent from our study, can be explained by several factors. First, the Polish people found themselves confronted by a crisis of a type completely unknown to them, and in such situations, people are characterized by conformism, submitting to the instructions of those who, in their opinion, know better [43]. Second, when the first Polish cases were detected, the media had already covered dramatic accounts of the fight against the new coronavirus in other countries (most prominently, Italy), which was also likely to have affected attitudes. Third, the tendency to complain is deeply rooted in Polish culture [44], and this might have caused highly pessimistic perceptions of the coronavirus-related situation. International comparisons made in mid-May 2020 showed that, among other differences, Poles differed from people of other European nations in their belief that the SARS-CoV-2 crisis would worsen rather than weaken [45].

Perhaps surprisingly, we did not identify a cluster of people who displayed little fear of the coronavirus, who believed that the restrictions introduced were unnecessary and did not comply with them. This may be attributable to the fact that the study was carried out at the very beginning of the pandemic. Nevertheless, the data confirmed the existence of a surprisingly widespread social mobilization in response to the virus, despite relatively critical perceptions of the actions taken by the Polish government. The previously quoted international data showed that in the first half of May (i.e., shortly after the date of our survey), less than 40% of Poles thought that the Polish government was coping well with the situation brought about by the pandemic. Only the French rated their government worse [45]. In the context of these results, and given that political divisions are highly visible on an everyday basis in Polish society, it is worth emphasizing that our study found that political views were not strongly related to attitudes toward the pandemic, although supporters of the party currently in power were slightly more likely to be characterized by an involved attitude than supporters of the main opposition party (53.9 vs. 48.4% respectively).

It is worth emphasizing that people with an involved attitude generally claimed strict adherence to government guidelines, but tended to admit that this was not the case during Easter. This can be explained by the fact that the Poles are one of the most religious nations in Europe [46], and Easter, the day of the Lord’s resurrection, is the most important holiday for Polish Christians in the whole liturgical year. So, it is unsurprising that even people with an involved attitude slightly lowered their level of submission to the national rules to adhere to the rites and traditions of Easter. People with other attitudes cared less about the restrictions than “involved” people, so their level of agreement to the statement: “At Easter, I adhered less strictly to the recommendations about not leaving home and not meeting other people” was lower. Our bivariate analysis showed that people who participated most frequently in religious rituals mostly displayed a cautious attitude (51%; other people being split equally among the other two attitudes, 24.5% each). Restrictions imposed by the state, which included restrictions on participating in religious rituals, were certainly difficult for such people, and the Polish media reported violations of government restrictions in connection with religious rituals [47].

Women were more likely to display an involved attitude than men (53.9 vs. 41.6%), slightly less likely to belong to the cautious attitudinal group (26.6 vs. 28.2%), and much less likely to be in the indifferent group (19.5 vs. 30.1%). Gender differences in attitudes (greater male indifference and lesser involvement) remained significant when other socio-demographic factors were included in logistic regression analyses. All these chime with other research on attitudes toward COVID-19 showing that females perceive greater vulnerability [27], perceive the threat as higher [48], and, particularly consistent with our results, are more likely to adopt preventive behaviors [48] and have a greater tendency to comply with quarantine orders [18]. These observations are in line with research showing that men are more likely to engage in risky activities that threaten their health, while women are more conservative [49,50]. However, it is worth remembering that in our study, a propensity to take risks was not significantly associated with the type of attitude displayed, the distributions of risk-taking (the minority) and more conservative (the majority) people being almost identical across the attitudinal groups.

Men are also more likely than women to engage in unhealthy behavior such as smoking, drug use, and alcohol abuse [49,50,51], and it is consistent with such behaviors that men should exhibit a more limited sense of threat to their health and lives during a pandemic. It is also worth noting that Polish society is quite traditional in its approach to the roles of women and men: women are usually responsible for household duties and taking care of the health of family members, including that of children and the elderly [52]. This may explain the greater sense of threat felt by women in relation to the virus in the context of concerns about mortality and health, especially with respect to members of their immediate families.

The World Health Organization’s website says that “Older people, and people with pre-existing medical conditions (such as asthma, diabetes, heart disease) appear to be more vulnerable to becoming severely ill with the virus” [53]. These are well-known facts, so it is not surprising that, although age differences in attitudes were not statistically significant, the oldest people in our study tended to be characterized by an involved attitude (50–59 years, 54.7%; 60 years and older, 57.1%). This is in line with previous COVID-19 research of Perrotta et al. [48], showing older respondents to have higher levels of awareness and concern. The majority of people assessing their health as poor were also in the involved attitudinal group (56.1%). The less dynamic transmission of the new coronavirus in Poland can also be explained by a cultural norm of taking care of the elderly [7] and the underdeveloped structure of the Polish elderly care industry [54], this generally placing the responsibility of care for the elderly on the family.

Although education was not one of the socio-demographic variables that significantly differentiated Poles’ attitudes towards the pandemic, it is worth noting that over 53% of people with a higher education displayed an involved attitude, this percentage being greater than that for less well-educated people. This is unsurprising given that the positive relationship between the level of education and health is well described in the scientific literature [55,56]. People who are better educated enjoy good health for longer than those who are less well educated; this is explained by differences in factors such as (1) working conditions and economic conditions, (2) socio-psychological resources, and (3) the tendency to have a healthy lifestyle [55]. With respect to the latter, a better education is associated with more rational choices concerning one’s own health and greater commitment to maintaining one’s health, an observation that is consistent with the previously mentioned Italian findings showing more highly educated people to be particularly likely to comply with quarantine orders [18].

Both bivariate and multivariate analyses identified a relationship between attitudes and respondents’ financial situations. A better financial situation accompanied less involvement in fighting the virus. An unfavorable financial situation results in less stability in life, and obeying the restrictions might have given people a greater sense of security. Although bivariate analysis showed no relationship between attitudes and (population) size of places where people resided, logistic regression analysis showed that a cautious attitude was more likely to occur among people living in smaller places. This may be because there is stronger social control in smaller social groups (more people know each other), and this may result in a reluctance to violate or bend established norms even where people are not completely sure of whether these norms are right or wrong.

Finally, there is previous evidence that life satisfaction is conducive to better health [57]. Therefore, it was no surprise to find that the logistic regression analysis showed that people who indicated satisfaction with their lives had a greater tendency to exhibit an involved attitude than those indicating less satisfaction.

Polish people’s attitudes (in particular, the absence of attitudes indicating an extremely dismissive posture toward COVID-19) constitute one explanation as to why the virus spread more slowly in Poland than in many other countries during the first wave of the pandemic. But, of course, other factors might also have played a role. Some scientists have examined factors to explain why some countries have been better at reducing the impact of the epidemic than others, which are completely independent of current human behavior patterns such as differences in conformity to social distancing guidelines, e.g., the impact of vaccination against tuberculosis on prevention of local COVID-19 spread [58], atmospheric pollution [59] and demographic factors such as differing numbers of older people (who are most at risk of developing acute symptoms and dying) [60].

With the passage of time, societal fatigue with the limitations has occurred, and there has been a change in Polish people’s attitudes. As part of this change, various public platforms are being used to promulgate the idea that the virus is just a fabrication and an anti-mask movement has appeared. Perhaps not surprisingly then, the virus spread rapidly during the second wave in autumn 2020, and, as of 8 January 2021, there were 36,104 confirmed cases per million inhabitants, and 30,574 deaths had occurred [4]. Although, this situation was still better than in the USA, where there were 66,675 cases per million, it was little different from that in some other European countries. For example, in terms of cases per million, the UK had 42,447 cases, Italy 36,752 cases, and Slovakia 36,836 cases [4].

Our study has limitations. Internet research carried out using CAWI techniques provides faster results than research conducted using face-to-face interviews, and this is particularly advantageous during an important and critical period such as that surrounding the COVID-19 pandemic, especially given the impossibility of face-to-face studies because of the restrictions introduced. Nevertheless, CAWI methods of data collection have disadvantages. In particular, it is difficult to reach all members of society. While around 70% of the Polish population have declared that they regularly use the Internet [61], this means that a substantial minority of Poles (people without Internet skills or access to the web) did not have the chance to air their opinions. Furthermore, using an internet panel as a sampling frame is likely to result in a deficit of less-educated people. In our analyses, the only way to address educational issues was to stratify people into only two educational levels: people with and without higher education. The limited number of people with very low levels of education in our sample is likely to have resulted in a slight over-sampling of people with liberal views, and a slight under-representation of people with conservative leanings. Although the impact of this is difficult to estimate, this may have resulted in a slightly distorted picture of the distribution of attitudes in the whole Polish population. A further limitation of the study is the fact that our data only represent a snapshot taken at a certain point during a quickly changing situation. The epidemiological situation around the world is changing rapidly, and all contemporary studies of the present epidemic are likely to become out of date shortly after their execution. Additionally, it should be noted that surveys on topics such as that presently studied cannot be guaranteed to be free from the effects of socially desirable responding. Finally, our research considered only one country, and a comparative international study would help to place our findings in a broader cultural context.

## 5. Conclusions

Cluster analysis allowed us to distinguish three types of attitudes adopted by people during the imposition of the most severe restrictions used to fight the first wave of the COVID-19 pandemic in Poland. We labeled the most common attitude “involved” (48.1%); this described people who were afraid of the virus, mainly for health and existential reasons, and who rigorously followed government guidelines. The other two attitudes were of similar prevalence: “cautious” people (27.4%) feared the virus more for material and professional reasons, although they complied with most guidelines, whereas “indifferent” people (24.6%) were not afraid for their own health or the health of their loved ones and were indifferent to the restrictions imposed; they selectively complied with the restrictions and believed they should be ended. The most important conclusion to be drawn from the research is that it did not identify any extreme attitude signaling a desire to ignore quarantining orders and other rules put in place by the Polish government. The widespread compliance of members of Polish society with the restrictions imposed at the time data were collected is one factor that may help to explain why the pandemic did not develop as dynamically in Poland as it did in many other countries during the spring of 2020. The study’s results illustrate the important role that social attitudes can play with respect to the public health issues which arise during a pandemic. Recognition of people’s attitudes is important for politicians and public health specialists as it can facilitate health crisis management. In particular, identifying socio-demographic differences in attitudes can facilitate the effective targeting of educational health promotion interventions. Our study makes an important contribution to the knowledge of Poles’ attitudes during the first wave of the pandemic and appears to be the first study to have used cluster analysis to distinguish Poles’ attitudes toward COVID-19 at this point in the pandemic’s time course.

## Figures and Tables

**Table 1:** Percentage of affirmative answers (Definitely yes and Probably yes) for the three attitude types.

Statement	Focus		
	1	2	3
	Type of Attitude		
	Indifferent (*n* = 246)	Involved (*n* = 481)	Cautious (*n* = 274)
	Percentage of Affirmative Answers		
I feel fear for my health	19.9	79.0	77.4
I feel fear for the health and life of my loved ones	40.2	89.6	83.2
I am afraid that I will be financially broken by the prolonged pandemic	32.5	65.7	77.7
I am afraid of losing my job because of the situation	27.2	51.6	67.5
The prolonged period of social isolation is negatively affecting my mental well-being	45.1	73.4	81.8
The restrictions introduced by the government in the fight against the pandemic are too strict	35.8	10.8	52.9
People leaving the house for a walk are acting irresponsibly in the present situation	23.2	61.1	51.1
The period of social restrictions imposed by the government in the fight against the virus should not be extended any further	31.7	11.9	52.6
I believe that, for the good of the economy, decisions should not be taken to close borders and many institutions	27.2	8.7	55.8
The global economy will recover quickly after the pandemic has been fought	28.9	22.2	43.8
The media have unnecessarily spread panic in society by exaggerating the situation	54.1	37.6	66.4
Thanks to the pandemic, people will understand what is really important in life	29.7	66.9	64.6
I strictly adhere to the restrictions imposed by the government in the fight against the pandemic	43.5	96.3	77.7
I have acquired appropriate food supplies to allow myself to stay at home for a long period of time	31.3	75.5	70.8
I have been wearing a mask for a long time when leaving the house	18.3	69.0	67.9
In the current situation, I would not offer my hand ^1^ to greet anyone except members of my household	30.9	84.8	73.4
I meet my friends and family outside my household quite regularly	13.4	1.9	33.6
I follow information about the pandemic daily, and monitor incidence statistics	36.2	87.9	73.0
If I developed coronavirus symptoms, I would immediately contact the appropriate infectious disease hospital or sanitary department	53.3	94.8	82.5
I would get vaccinated if a coronavirus vaccine was already available	29.7	76.3	65.3
I am now trying to take care of my immunity better by engaging in appropriate healthy behavior	32.5	74.6	80.7
I try to go for a walk regularly or do other outdoor activities	33.3	20.0	58.0
At Easter, I adhered less strictly to the recommendations about not leaving home and not meeting other people	26.0	12.5	54.4
I know exactly what to do if I observe coronavirus symptoms in myself or members of my household	48.8	80.9	71.5
Coronavirus is nothing more than a worse type of flu	33.3	16.4	52.9

^1^ This is a gesture used when saying “hello” in Poland, especially among men. Shadings indicate strengths of loadings for each cluster as follows: white, weakest loadings; light grey, moderate loadings; dark grey, strongest loadings.

**Table 2:** Socio-demographic characteristics of people falling into the different clusters.

**Profile Characteristics**	Cluster 1 Indifferent (*n* = 246)		Cluster 2 Involved (*n* = 481)		Cluster 3 Cautious (*n* = 274)		Statistics		Holm-Bonferroni Correction *p*
	*n*	%	*n*	%	*n*	%	Cramer’s *V*	*p*	
**Gender**							0.140	**<0.001**	**<0.001**
**Female**	102	19.5%	282	53.9%	139	26.6%			
**Male**	144	30.1%	199	41.6%	135	28.2%			
**Age**							0.065	0.381	0.762
**18–29**	75	24.2%	144	46.5%	91	29.4%			
**30–39**	73	26.9%	120	44.3%	78	28.8%			
**40–49**	46	24.9%	86	46.5%	53	28.6%			
**50–59**	32	23.4%	75	54.7%	30	21.9%			
**60+**	20	20.4%	56	57.1%	22	22.4%			
**Education**							0.074	**0.026**	0.130
**Primary, lower secondary, vocational**	34	28.3%	52	43.3%	34	28.3%			
**Secondary education**	119	23.9%	224	45.0%	155	31.1%			
**Higher education**	93	24.3%	205	53.5%	155	22.2%			
**Place of residence**							0.085	0.070	0.280
**Village**	84	24.5%	143	41.7%	116	33.8%			
**City of up to 19,999**	30	26.1%	55	47.8%	30	26.1%			
**City 20,000–199,999**	44	21.9%	107	53.2%	50	24.9%			
**City 200,000–499,999**	55	27.0%	104	51.0%	45	22.1%			
**City of over 500,000**	33	23.9%	72	52.2%	33	23.9%			
**Frequency of participation in religious practices**							0.126	**<0.001**	**<0.001**
**Several times a week**	12	24.5	12	24.5	25	51.0			
**Once a week**	77	26.6	140	49.7	69	23.8			
**1-2 times a month**	24	20.7	45	38.8	47	40.5			
**Several times a year**	50	22.5	116	52.3	56	25.2			
**Once every few years**	20	24.4	44	53.7	18	22.0			
**Not at all**	63	26.0	120	49.6	59	24.4			
**Financial situation**							0.112	**0.001**	**0.007**
**Very good**	28	31.8	22	25.0	38	43.2			
**Good**	90	24.4	178	48.2	101	27.4			
**Moderate**	103	22.6	237	52.0	116	25.4			
**Poor**	18	26.1	36	52.2	15	21.7			
**Very poor**	7	36.8	8	42.1	4	21.1			
**Self-assessment of health**							0.096	**0.005**	**0.030**
**Very good**	78	27.6%	117	41.3%	88	31.1%			
**Good**	97	20.2%	248	51.6%	136	28.3%			
**Moderate**	62	31.6%	93	47.4%	41	20.9%			
**Bad**	9	22.0%	23	56.1%	9	22.0%			
**Life satisfaction**							0.065	0.074	0.280
**Satisfied**	170	22.5%	378	50.1%	206	27.3%			
**Dissatisfied**	48	29.4%	68	41.7%	47	28.8%			
**Hard to say**	28	33.3%	35	41.7%	21	25.0%			
**Propensity to take risks**							0.009	0.963	0.963
**Yes**	72	24.2%	142	47.8%	83	27.9%			
**No**	174	24.7%	339	48.2%	191	27.1%			
**Political preference**							0.134	**<0.001**	**<0.001**
**EC Law and Justice –** **ruling party**	49	20.2%	131	53.9%	63	25.9%			
**EC Civic Coalition –** **main opposition**	27	17.4%	75	48.4%	53	34.2%			
**EC Democratic Left Alliance**	28	24.1%	60	51.7%	28	24.1%			
**EC Freedom and Independence Confederation**	34	36.2%	24	25.5%	36	38.3%			
**EC Polish People’s Party**	16	24.6%	30	46.2%	19	29.2%			
**Other**	3	20.0%	9	60.0%	3	20.0%			
**None**	40	30.5%	59	45.0%	32	24.4%			
**Hard to say**	49	26.9%	93	51.1%	40	22.0%			

Note: Bold font indicates statistical significance. An alternative analysis was performed adopting a different method of calculating percentages: in this analysis, percentages for categories within each socio-demographic variable summed to 100% within each attitude column separately (i.e., percentages were calculated for each socio-demographic variable within columns rather than percentages being calculated across attitude columns). The relevant table and its interpretation are presented in Appendix D.

**Table 3:** This is a table. Tables should be placed in the main text near to the first time they are cited.

Independent (Categorical) Variable:	Dependent variable								
	Analysis 1 Indifferent vs. Others			Analysis 2 Involved vs. Others			Analysis 3 Cautious vs. Others		
	Odds Ratio	95% CI	*p*	Odds Ratio	95% CI	*p*	Odds Ratio	95% CI	*p*
**Gender** (F–M)	1.769	(1.32–2.38)	<0.001	0.624	(0.48– 0.81)	<0.001	1.045	(0.79– 1.39)	0.759
**Age** (ascending)	0.960	(0.85– 1.09)	0.518	1.039	(0.93– 1.15)	0.479	0.991	(0.88–1.12)	0.885
**Education** (ascending)	0.983	(0.87– 1.11)	0.778	1.109	(1.00–1.24)	0.060	0.899	(0.80– 1.01)	0.076
**Size of place of residence** (ascending)	1.030	(0.93– 1.14)	0.578	1.074	(0.98– 1.17)	0.118	0.892	(0.81– 0.99)	0.026
**Frequency of participation in religious practices** (ascending)	1.011	(0.92– 1.11)	0.813	0.940	(0.87–1.02)	0.127	1.067	(0.98–1.17)	0.153
**Financial situation** (ascending)	1.152	(0.94– 1.41)	0.169	0.748	(0.63– 0.89)	0.001	1.243	(1.02–1.51)	0.029
**Self-assessment of health** (bad–good)	1.014	(0.82– 1.26)	0.896	0.866	(0.72– 1.05)	0.135	1.181	(0.96–1.46)	0.123
**Life satisfaction** (ascending)	0.809	(0.69–0.95)	0.011	1.282	(1.10–1.49)	0.001	0.911	(0.77–1.07)	0.262
**Propensity to take risks** (no–yes)	1.018	(0.74–1.40)	0.916	0.912	(0.69–1.21)	0.520	1.104	(0.81–1.50)	0.531
**Constant**	0.179		0.011	2.335		0.154	0.187		0.011

Note: Shadings indicate indicates were is statistical significance. Political preference was not included in the regression models because of its measurement level.

## Data Availability

Data available in a publicly accessible repository. The data presented in this study are openly available in figshare at https://figshare.com/articles/Poland_-_COVID-19/12547337.

## Appendix A

**Table A1:** Distribution of socio-demographic characteristics in the studied sample.

Profile Characteristics	*n*	%
Gender		
**Female**	523	52.2
**Male**	478	47.8
Age		
**18–29**	310	31.0
**30–39**	271	27.1
**40–49**	185	18.5
**50–59**	137	13.7
**60+**	98	9.8
Education		
**Primary, lower secondary, vocational**	120	12.0
**Secondary education**	498	49.8
**Higher education**	383	38.3
Place of residence		
**Village**	343	34.3
**City of up to 19,999**	115	11.5
**City 20,000–199,999**	201	20.1
**City 200,000–499,999**	204	20.4
**City of over 500,000**	138	13.8
Frequency of participation in religious practices		
**Several times a week**	49	4.9
**Once a week**	290	29.0
**1–2 times a month**	116	11.6
**Several times a year**	222	22.2
**Once every few years**	82	8.2
**Not at all**	242	24.2
Financial situation		
**Very good**	88	8.8
**Good**	369	36.9
**Moderate**	456	45.6
**Poor**	69	6.9
**Very poor**	19	1.9
Self-assessment of health		
**Very good**	283	28.3
**Good**	481	48.1
**Moderate**	196	19.6
**Bad**	41	4.1
Life satisfaction		
**Satisfied**	754	75.3
**Dissatisfied**	163	16.3
**Hard to say**	84	8.4
Propensity to take risks		
**Yes**	297	29.7
**No**	704	70.3
Political preference		
**EC Law and Justice—ruling party**	243	24.3
**EC Civic Coalition—main opposition**	155	15.5
**EC Democratic Left Alliance**	116	11.6
**EC Freedom and Independence Confederation**	94	9.4
**EC Polish People’s Party**	65	6.5
**Other**	15	1.5
**None**	131	13.1
**Hard to say**	182	18.2

## Appendix B

**Table A2:** Final cluster centers resulting from cluster analysis using the k-means method (scale from –2 to +2).

Assessed Statements on the Scale from –2 (Strongly Disagree) to +2 (Strongly Agree):	Focus		
	1	2	3
	Type of Attitude		
	Indifferent (*n* = 246)	Involved (*n* = 481)	Cautious (*n* = 274)
	Ultimate Focus Centers		
I feel fear for my health	0	1	1
I feel fear for the health and life of my loved ones	0	1	1
I am afraid that I will be financially broken by the prolonged pandemic	0	1	1
I am afraid of losing my job because of the situation	0	0	1
The prolonged period of social isolation is negatively affecting my mental well-being	0	1	1
The restrictions introduced by the government in the fight against the pandemic are too strict	0	–1	0
People leaving the house for a walk are acting irresponsibly in the present situation	0	1	0
The period of social restrictions imposed by the government in the fight against the virus should not be extended any further	0	–1	0
I believe that, for the good of the economy, decisions should not be taken to close borders and many institutions	0	–1	0
The global economy will recover quickly after the pandemic has been fought	0	–1	0
The media have unnecessarily spread panic in society by exaggerating the situation	1	0	1
Thanks to the pandemic, people will understand what is really important in life	0	1	1
I strictly adhere to the restrictions imposed by the government in the fight against the pandemic	0	1	1
I have acquired appropriate food supplies to allow myself to stay at home for a long period of time	0	1	1
I have been wearing a mask for a long time when leaving the house	0	1	1
In the current situation, I would not offer my hand to greet anyone except members of my household	0	1	1
I meet my friends and family outside my household quite regularly	–1	–2	0
I follow information about the pandemic daily, and monitor incidence statistics	0	1	1
If I developed coronavirus symptoms, I would immediately contact the appropriate infectious disease hospital or sanitary department	1	2	1
I would get vaccinated if a coronavirus vaccine was already available	0	1	1
I am now trying to take care of my immunity better by engaging in appropriate healthy behavior	0	1	1
I try to go for a walk regularly or do other outdoor activities	0	–1	0
At Easter, I adhered less strictly to the recommendations about not leaving home and not meeting other people	0	–1	0
I know exactly what to do if I observe coronavirus symptoms in myself or members of my household	0	1	1
Coronavirus is nothing more than a worse type of flu	0	–1	0

## Appendix C

**Table A3:** Distributions of responses to the statements included in the cluster analysis.

Statement	Strongly Agree	Agree	Hard to Say	Disagree	Strongly Disagree
	Percentage of Affirmative Answers				
I feel fear for my health	22.8	41.3	17.5	14.2	4.3
I feel fear for the health and life of my loved ones	36.8	39.0	13.5	7.4	3.4
I am afraid that I will be financially broken by the prolonged pandemic	27.3	33.6	21.7	13.7	3.8
I am afraid of losing my job because of the situation	22.8	27.2	21.9	18.7	9.5
The prolonged period of social isolation is negatively affecting my mental well-being	30.5	38.3	14.5	13.0	3.8
The restrictions introduced by the government in the fight against the pandemic are too strict	9.4	19.1	23.5	30.7	17.4
People leaving the house for a walk are acting irresponsibly in the present situation	20.7	28.4	27.4	18.3	5.3
The period of social restrictions imposed by the government in the fight against the virus should not be extended any further	10.3	17.6	33.5	26.3	12.4
I believe that, for the good of the economy, decisions should not be taken to close borders and many institutions	8.8	17.4	27.7	27.9	18.3
The global economy will recover quickly after the pandemic has been fought	5.3	13.3	34.5	34.4	12.6
The media have unnecessarily spread panic in society by exaggerating the situation	21.0	28.6	24.9	18.5	7.1
Thanks to the pandemic, people will understand what is really important in life	20.9	36.3	25.1	12.7	5.1
I strictly adhere to the restrictions imposed by the government in the fight against the pandemic	31.5	46.8	14.0	5.9	1.9
I have acquired appropriate food supplies to allow myself to stay at home for a long period of time	22.8	40.6	13.7	17.6	5.4
I have been wearing a mask for a long time when leaving the house	22.1	34.2	13.2	22.2	8.4
In the current situation, I would not offer my hand to greet anyone except members of my household	37.8	30.7	18.8	9.2	3.6
I meet my friends and family outside my household quite regularly	4.3	9.1	11.5	30.0	45.2
I follow information about the pandemic daily, and monitor incidence statistics	30.9	40.3	13.4	11.2	4.3
If I developed coronavirus symptoms, I would immediately contact the appropriate infectious disease hospital or sanitary department	49.6	31.7	14.0	3.1	1.7
I would get vaccinated if a coronavirus vaccine was already available	33.5	28.4	22.1	8.3	7.8
I am now trying to take care of my immunity better by engaging in appropriate healthy behavior	21.2	44.8	21.6	10.5	2.0
I try to go for a walk regularly or do other outdoor activities	9.7	24.0	18.3	30.5	17.6
At Easter, I adhered less strictly to the recommendations about not leaving home and not meeting other people	10.6	16.7	13.4	22.8	36.6
I know exactly what to do if I observe coronavirus symptoms in myself or members of my household	27.6	42.9	20.4	6.8	2.4
Coronavirus is nothing more than a worse type of flu	8.3	22.3	25.7	26.4	17.4

## Appendix D

**Table A4:** A different method of calculating percentages.

	Cluster 1 Indifferent (*n* = 246)		Cluster 2 Involved (*n* = 481)		Cluster 3 Cautious (*n* = 274)		Statistics		
Profile Characteristics	*n*	%	*n*	%	*n*	%	Cramer’s *V*	*p*	Holm–Bonferroni Correction *p*
**Gender**							0.140	**<0.001**	**<0.001**
**Female**	102	41.5	282	58.6	139	50.7			
**Male**	144	58.5	199	41.4	135	49.3			
		100.0		100.0		100.0			
**Age**							0.065	0.381	0.762
**18–29**	75	30.5	144	29.9	91	33.2			
**30–39**	73	29.7	120	24.9	78	28.5			
**40–49**	46	18.7	86	17.9	53	19.3			
**50–59**	32	13.0	75	15.6	30	10.9			
**60+**	20	8.1	56	11.6	22	8.0			
		100.0		100.0		100.0			
**Education**							0.074	**0.026**	0.130
**Primary. lower secondary. vocational**	34	13.8	52	10.8	34	12.4			
**Secondary education**	119	48.4	224	46.6	155	56.6			
**Higher education**	93	37.8	205	42.6	155	31.0			
		100.0		100.0		100.0			
**Place of residence**							0.085	0.070	0.280
**Village**	84	34.1	143	29.7	116	42.3			
**City of up to 19,999**	30	12.2	55	11.4	30	10.9			
**City 20,000–199,999**	44	17.9	107	22.2	50	18.2			
**City 200,000–499,999**	55	22.4	104	21.6	45	16.4			
**City of over 500,000**	33	13.4	72	15.0	33	12.0			
		100.0		100.0		100.0			
**Frequency of participation in religious practices**							0.126	**< 0.001**	**<0.001**
**Several times a week**	12	4.9	12	2.5	25	9.1			
**Once a week**	77	31.3	140	29.9	69	25.2			
**1–2 times a month**	24	9.8	45	9.4	47	17.2			
**Several times a year**	50	20.3	116	24.1	56	20.4			
**Once every few years**	20	8.2	44	9.1	18	6.6			
**Not at all**	63	25.6	120	24.9	59	21.5			
		100		100		100			
**Financial situation**							0.112	0.001	**0.007**
**Very good**	28	11.4	22	4.6	38	13.9			
**Good**	90	36.6	178	37.0	101	36.9			
**Moderate**	103	41.9	237	49.3	116	42.3			
**Poor**	18	7.3	36	7.5	15	5.5			
**Very poor**	7	2.8	8	1.7	4	1.5			
		100.0		100.0		100.0			
**Self-assessment of health**							0.096	**0.005**	**0.030**
**Good or very good**	175	71.1	365	75.9	224	81.7			
**Moderate**	62	25.2	93	19.3	41	15.0			
**Bad**	9	3.7	23	4.8	9	3.3			
		100.0		100.0		100.0			
**Life satisfaction**							0.065	0.074	0.280
**Satisfied**	170	69.1	378	78.6	206	75.2			
**Dissatisfied**	48	19.5	68	14.1	47	17.2			
**Hard to say**	28	11.4	35	7.3	21	7.7			
		100.0		100.0		100.0			
**Propensity to take risks**							0.009	0.963	0.963
**Yes**	72	29.3	142	29.5	83	30.3			
**No**	174	70.7	339	70.5	191	69.7			
		100.0		100.0		100.0			
**Political preference**							0.134	**<0** **.** **001**	**<0.001**
**EC Law and Justice –** **ruling party**	49	19.9	131	27.2	63	23.0			
**EC Civic Coalition –** **main opposition**	27	11.0	75	15.6	53	19.3			
**EC Democratic Left Alliance**	28	11.4	60	12.5	28	10.2			
**EC Freedom and Independence Confederation**	34	13.8	24	5.0	36	13.1			
**EC Polish People’s Party**	16	6.5	30	6.2	19	6.9			
**Other**	3	1.2	9	1.9	3	1.1			
**None**	40	16.3	59	12.3	32	11.7			
**Hard to say**	49	19.9	93	19.3	40	14.6			
		100.0		100.0		100.0			

Use of a different method of calculating percentages, in which the percentages were calculated for each socio-demographic variable within columns (rather percentages being calculated across attitude columns), showed that people with an indifferent attitude were more likely to be described by the following attributes relative to those displaying the other attitudes: male gender, an age of 30–39 years, a low level of education, resident in a village, a regular religious practitioner, a good or moderate financial situation, moderate self-assessed health, a lack of satisfaction with life, and support for the ruling party or lacking a specific party sympathy.

Relative to people with the other attitudes, people with an involved attitude were slightly more likely to be characterized by features such as female gender, an age of 50+ years, a higher education, residence in a medium or large city, rare engagement in/complete abstinence from religious practice, a good or average financial situation, satisfaction with life, and support for either the right-leaning ruling party or its left-leaning opposition.

People with a cautious attitude did not differ in terms of gender, but were relatively more likely to have the following attributes: an age of 18–29 years, educated to secondary level, residence in the countryside, regular engagement in religious practices, a moderate, good, or very good financial situation, satisfaction with health, support for the ruling party or the main opposition faction: the Civic Coalition.
